# Unveiling Usage Patterns and Explaining Usage of Symptom Checker Apps: Explorative Longitudinal Mixed Methods Study

**DOI:** 10.2196/55161

**Published:** 2024-12-09

**Authors:** Anna-Jasmin Wetzel, Christine Preiser, Regina Müller, Stefanie Joos, Roland Koch, Tanja Henking, Hannah Haumann

**Affiliations:** 1 Institute of General Practice and Interprofessional Care University Hospital Tübingen Tübingen Germany; 2 Institute of Occupational and Social Medicine and Health Services Research University Hospital Tübingen Tübingen Germany; 3 Institute for Philosophy University of Bremen Bremen Germany; 4 Institute for Applied Social Sciences Technical University of Würzburg-Schweinfurt Würzburg Germany

**Keywords:** self-triage, eHealth, self-diagnosis, mHealth, mobile health, usage, patterns, predicts, prediction, symptoms checker, apps, applications, explorative longitudinal study, self care, self management, self-rated, mixed method, circumstances, General Linear Mixed Models, GLMM, qualitative data, content analysis, Kuckartz, survey, participants, users

## Abstract

**Background:**

Symptom checker apps (SCA) aim to enable individuals without medical training to classify perceived symptoms and receive guidance on appropriate actions, such as self-care or seeking professional medical attention. However, there is a lack of detailed understanding regarding the contexts in which individuals use SCA and their opinions on these tools.

**Objective:**

This mixed methods study aims to explore the circumstances under which medical laypeople use SCA and to identify which aspects users find noteworthy after using SCA.

**Methods:**

A total of 48 SCA users documented their medical symptoms, provided open-ended responses, and recorded their SCA use along with other variables over 6 weeks in a longitudinal study. Generalized linear mixed models with and those without regularization were applied to consider the hierarchical structure of the data, and the models’ outcomes were evaluated for comparison. Qualitative data were analyzed through Kuckartz qualitative content analysis.

**Results:**

Significant predictors of SCA use included the initial occurrence of symptoms, day of measurement (odds ratio [OR] 0.97), self-rated health (OR 0.80, *P*<.001), and the following International Classification in Primary Care-2–classified symptoms, that are general and unspecified (OR 3.33, *P*<.001)*,* eye (OR 5.56, *P*=.001), cardiovascular (OR 8.33, *P*<.001), musculoskeletal (OR 5.26, *P*<.001), and skin (OR 4.76, *P*<.001). The day of measurement and self-rated health showed minor importance due to their small effect sizes. Qualitative analysis highlighted four main themes: (1) reasons for using SCA, (2) diverse affective responses, (3) a broad spectrum of behavioral reactions, and (4) unmet needs including a lack of personalization.

**Conclusions:**

The emergence of new and unfamiliar symptoms was a strong determinant for SCA use. Specific International Classification in Primary Care–rated symptom clusters, particularly those related to cardiovascular, eye, skin, general, and unspecified symptoms, were also highly predictive of SCA use. The varied applications of SCA fit into the concept of health literacy as bricolage, where SCA is leveraged as flexible tools by patients based on individual and situational requirements, functioning alongside other health care resources.

## Introduction

Symptom checker apps (SCA) are an emerging class of health technology tools that enable patients to input symptoms and biodata to receive a list of probable diagnoses and related triage suggestions [1]. Research on SCAs has largely focused on their accuracy, for instance, a study by Semigran et al [2] evaluated 23 SCAs using patient vignettes, revealing that the correct diagnosis appeared as the most probable in only 34% of cases. The appropriateness of the triage advice was found to be satisfactory in 57% of cases [2]. Notably, the accuracy of SCAs in nonemergency situations decreased as the urgency of the presented scenarios decreased [2]. A subsequent audit in 2022 indicated that there had been no significant improvement in triage performance over the past 5 years, with considerable variability in effectiveness among different SCAs [3]. Comparisons between SCAs and the decision-making of nonprofessionals showed no superiority in triage capability by SCAs [4]. Another study using simulated patients’ cases showed that SCAs correctly identified the primary diagnosis in 30% of cases, significantly less than emergency physicians, who had an 80% success rate [5]. Despite a slightly better performance in emergency triage situations [4], physicians still vastly outperformed SCAs, with a 96% accuracy rate compared with 71% for SCAs [5]. Users of SCAs are typically characterized as younger females with a higher socioeconomic status [6] and a greater affinity for technology [7]. In addition, it has been observed that SCA users showed higher levels of health anxiety than nonusers [8], with SCAs being more widely recognized among these users [6]. Users with anxiety disorders, however, tend to find SCAs less beneficial [6], indicating that SCAs might not be advantageous for this user group. The literature suggests that such patient groups may not only fail to benefit from using SCAs but could also experience heightened anxiety as a result, implying that it might be better to avoid these applications [9].

The impact of SCAs on health care providers is also critical, as patients may use these tools as a substitute for or in addition to professional health care services [10]. The level of trust users place in SCAs and their previous medical knowledge significantly affects how SCA results are interpreted [11,12]. SCA might also lead to the inefficient use of health care resources [5], as users may seek emergency department care too soon or too frequently. This behavior, especially from those with conditions that are not urgent, can intensify the burden on health care systems by potentially increasing costs and reallocating resources away from individuals who require immediate medical attention [13,14]. This outcome contrasts with the promises that SCAs would reduce pressure on health care systems. Currently, it is unclear whether SCAs have an impact on health care systems and, if so, the nature of this impact.

The factors driving SCA usage remain underexplored. Research has shown that gender, age, health anxiety, and other psychosocial factors significantly influence SCA use [6]. User characteristics, including health anxiety, acceptance of technology, and education level, also affect how SCAs are used [15,16]. Despite this knowledge, there is a notable gap in research regarding the situational factors that prompt SCA usage. Gaining a deeper insight into these situational factors that lead to SCA use is essential for comprehending the broader implications of SCA use on both individuals and health care providers. Consequently, this study uses a mixed methods approach to explore how medical laypersons use SCAs, aiming to understand the patterns of use and the users’ perceptions following their experience with a symptom checker.

## Methods

### Overview

This study was conducted using a mixed methods study approach, incorporating both qualitative and quantitative components within a prospective longitudinal cohort study design. The primary outcome of this study was whether one specific SCA was used during a day or not by Ada [17]. Ada [17] was chosen for the study due to its stage of development and its professed broad usage. Moreover, the application is ISO (International Organization for Standardization)-certified and possesses the CE mark. The use of one specific SCA was analyzed to ensure standardized interaction with one SCA only. The STROBE (The Strengthening the Reporting of Observational Studies in Epidemiology) guidelines were applied [18]. This study was conducted as a part of the joint project CHECK.APP [19].

In order to answer the research questions (predictors and usage patterns of SCA, aspects noteworthy after usage) of this mixed methods study, different longitudinal mixed models for SCA use were explored and compared.

### Ethical Considerations

Ethical approval was granted by the ethics committee of the medical faculty of the University of Tuebingen (464/202BO). Each participant was compensated 300€ (US $358) for their participation in the study. In accordance with the ethical approval, all participants provided written consent before taking part in the user diaries. A written and verbal explanation was provided, and participation in the study was voluntary.

### Measurements

User diaries were filled out daily by the study participants over a period of 6 weeks. SCA use was binary coded (yes or no). Further, the user diary comprised a survey that contained different questions about self-rated mood, self-rated health, self-rated stress level, and occurrence of physical symptoms throughout the day. Self-rated health and mood were both assessed with a visual analog 5-point scale. Subjectively rated stress was assessed with a single-choice item and 3 answer possibilities. If participants had symptoms, they stated what kind of symptoms they had, whether they took medication, and if they used a specific SCA [17]. If participants used the SCA on a day this was classified as a use-case, if the SCA was not used on a day this was classified as a nonuse case. Participants were asked if the symptoms appeared for the first time. Those who reported symptoms were asked to describe them in open-ended responses. After the data acquisition, reported symptoms were classified according to the International Classification in Primary Care 2 (ICPC) by a doctor from the study team, using dummy coding (present not present). Age was assessed in years, and gender was assessed as an open-ended response resulting in the 2 categories “male” and “female.” The variable “day of study” was retrieved from the dates in the user diaries.

Further questions considering the procedure after the app use were recorded as well. If the SCA was used, open-ended responses in the user diary asked for the experience with the SCA.

### Data Collection

The participants of the study were recruited from December 2020 to March 2021, with data collection taking place from April 2021 to September 2021. Due to the exploratory nature of the study, the sample size was determined based on estimated effect sizes and the feasibility of data collection. The requirement of the ethics committee for the study was not to initiate the use of SCA, and therefore only to include participants in the study who were already using SCA before the study. The participants were recruited from a previously conducted survey of the CHECK.APP project, specifically those who reported using a specific SCA [17]. The recruitment process for the survey involved using mailing lists from the University of Tuebingen, a social media campaign by the University Hospital Tuebingen, and social media channels from a statutory health insurance (Allgemeine Ortskrankenkasse Baden-Wuerttemberg). Participants were then contacted through the mail and asked whether they wanted to participate in a further study part.

Prior to the study’s start, web-based workshops were conducted to train participants on how to use the user diary and to enable them to ask questions to prevent common mistakes. Participants were directed to use the SCA as they normally would in their everyday activities. They chose when to start their 6-week period and submitted their diaries after finishing it. About 3 months after the workshop, they were sent reminder emails.

### Inclusion and Exclusion Criteria

The inclusion criteria for the study included having at least a B2 level of proficiency in the German language, as classified by the Common European Framework of Reference for Languages, an age older than 18 years, and being an Ada user. The exclusion criterion was possessing a medical degree.

### Data Analysis

#### Quantitative Data

To analyze and process the quantitative data, R statistics (version 4.1.3; R Core Team) [20] and RStudio (version 1.4) [20] were used. The aim of the present mixed methods study is to investigate the context in which SCAs are used by medical laypeople. To reach this aim, different longitudinal mixed models for SCA use are explored and compared.

#### Generalized Linear Mixed Model

To consider the hierarchical data generalized linear mixed models (GLMMs) with regularization, using a least absolute shrinkage and selection operator (LASSO) penalization, and without regularization were fit to the data and compared. The R package glmmPen [21] was used to conduct the LASSO penalized GLMM, and unpenalized model parameters were derived using the R package lme4 [22]. Further explanation can be found in Multimedia Appendix 1 and Textbox 1.

The model assessment was realized by using the Bayesian information criterion (BIC) scores of the models.

If model 3 or 4 would be identified as best performing they were fit with a GLMM without regularization to derive parameter estimators, CI, and SE.

**Mixed models.**

- Model 0: Intercept-only model, with a random intercept for participants
- Model 1: Generalized linear mixed models based on expert knowledge containing only fixed effects for the variables:
  Day of measurement
  First-time appearance of symptoms
  Self-rated health
- Model 2: as model 1 with added random effects for the variables “self-rated health,” “first time appearance of symptoms”
- Model 3: Regularized generalized linear mixed models with least absolute shrinkage and selection operator penalty containing all predictors of Table 1, with independent variance-covariance matrix
- Model 4: as model 3 with unstructured variance-covariance matrix

**Table 1:** Coefficients, CI, and *P* values for Model 0-3.

Coefficient	Model 0^a^			Model 1^b^			Model 2^c^			Model 3^d^	
	OR (95% CI)	*P* values	OR (95% CI)		*P* values	OR (95% CI)		*P* values	OR (95% CI)		*P* values
Intercept	0.14 (0.12-0.17)	<.001	3.49 (1.76-6.92)		<.001	5.18 (2.41-11.13)		<.001	359.87 (55.38-2338.31)		<.001
Day of measurement	—^e^	—	0.97 (0.96-0.98)		<.001	0.97 (0.96-0.98)		<.001	0.97 (0.96-0.99)		<.001
Self-rated health	—	—	0.44 (0.37-0.52)		<.001	0.40 (0.32-0.49)		<.001	0.80 (0.64-1.01)		<.001
Initial occurrence of Symptoms (yes)	—	—	9.28 (6.06- 14.22)		<.001	13.95 (6.20-31.37)		<.001	5.97 (3.67-9.72)		<.001
General and unspecified (present)	—	—	—		—	—		—	3.33 (1.92-5.56)		<.001
Eye (present)	—	—	—		—	—		—	5.56 (2.08-12.29)		<.001
Cardiovascular (present)	—	—	—		—	—		—	8.33 (2.70-25.00)		<.001
Musculoskeletal (present)	—	—	—		—	—		—	5.26 (3.44-8.33)		<.001
Skin (present)	—	—	—		—	—		—	4.76 (2.04-11.11)		<.001

^a^σ^2^=3.29; τ_00_=0.18_ID_; Bayesian information criterion=1568.02; intraclass correlation coefficient=0.05; N=48 (2016 observations).

^b^σ^2^=3.29; τ_00_=0.26_ID_; Bayesian information criterion=1306.17; intraclass correlation coefficient=0.07; N=48 (2016 observations).

^c^σ^2^=3.29; τ_00_=0.40_Intercept_; τ_11_=0.03 (participant-rated health) and 3.26 (first-time appearance of symptoms); ρ_01_=–0.65 and –0.06; Bayesian information criterion=1328.20; intraclass correlation coefficient=0.13; N=48 (2016 observations).

^d^σ^2^=3.29; τ_00_=1.14_Intercept_; τ_11_=1.53 (general and unspecified); ρ_01_=–0.89_ID_; Bayesian information criterion=1134.82; intraclass correlation coefficient=0.13; N=48 (2016 observations).

^e^Not applicable.

#### Missing Data

An Overview of missing data on the outcome and predictor variables can be found in Multimedia Appendix 2. All variables had less than 5% missing data. Missing data on the primary outcome and predictors were imputed using the R package missForest [23], that enables the imputation of missing in mixed data (categorical and continuous), based on a random forest approach [23]. The out-of-bag error (proportion of falsely classified) was used to quantify the test error.

#### Qualitative Data Analysis

The written comments from the open-ended questions were analyzed with qualitative content analysis [24]. Main categories and subcategories were built inductively after initial familiarization with the data [25]. All transcripts were analyzed by JW using MaxQDA [26]. For quality control, 40% of the codings were independently coded by CP. Afterward, the few conflicting codings were discussed by both coders in data sessions.

## Results

### Descriptive Data

Of the 48 participants, 31 (71%) were female. The mean age of the participants was 27 (SD 9.1, range 19-64) years. The primary outcome variable showed 264 use cases and 1752 nonuse cases. Imputation of missings resulted in an out-of-bag error of 0.063.

Symptoms of the chapter blood had low cell frequencies, the variable “Blood” was therefore excluded as a predictor in the following data analysis for the primary outcome. Tables with frequencies stratified by the outcome of the predictor variables can be found in Multimedia Appendices 3 and 4.

### Quantitative Results: Primary Outcome and Model Comparison

All assumptions of GLMM were met. Binned residual Plots for Models 1-3 can be found in Multimedia Appendix 5. The Null model revealed an intraclass correlation of 0.05 and indicated no substantial evidence of clustering between the participants. The BIC of the Intercept-only model was BIC=1568.02. Model 1 derived a BIC of 1305.00. The BIC achieved by Model 2 was higher (BIC=1326.76) compared with the BIC of Model 1. The BIC of Model 3 (BIC=1165.60) revealed the best model performance with the lowest value. Therefore, this model was chosen over Model 4 (BIC=1180.12) and was fit using a GLMM without regularization to derive parameter estimators, CI and SE.

LASSO regularization of Model 3 identified 8 fixed effect predictors, which are the day of measurement, self-rated health, the first-time appearance of symptoms, and 5 ICPC classified symptom clusters (general and unspecified, eye, cardiovascular, skin, and musculoskeletal) as meaningful. All predictors except self-rated health revealed a significant *P*<.001.

An overview of parameter estimations, 95% CI, and the BIC can be found in Table 1.

### Qualitative Results

The qualitative content analysis revealed four different main categories: (1) reasons for using SCA (n=79), (2) affective response and evaluation considering SCA usage (n=122), (3) Behavioral response considering SCA usage (n=66), and (4) unmet requirements (n=33).

As users made several entries into the diaries, topics varied between users in general and between the days the entry was made.

#### Reasons for Using Symptom Checker Apps

Reasons for SCA use emerged as a key topic. Participants identified several reasons for using SCA, including gaining information about medical conditions, symptoms, and treatment options. Participants also used symptom checkers to classify their symptoms and to understand the potential underlying causes of their health issues. The latter is not only to their own symptoms but also to the symptoms of close relatives and friends. Another stated reason for using a SCA was to determine whether a visit to a healthcare professional was necessary or not. Participants also reported using symptom checkers to cross-reference their own assumptions about their health concerns against the information provided by the SCA.

For validation: It's probably “really just” migraine & my head isn't about to burst!

In addition, SCA was used for verifying medical diagnoses and gaining a better understanding of one’s health concerns, which was seen as helpful in order to make informed decisions about one’s health and well-being. Moreover, participants used already known diseases or conditions to check if the SCA was able to detect them correctly and see how the SCA performed.

After the COVID-19 vaccination, I experienced side effects such as fever, headache, and dizziness. I knew that these symptoms were related to the vaccination, but I still wanted to know if the SCA could attribute these symptoms to the vaccination, which it did.

#### Affective Response and Evaluation Considering the Usage of Symptom Checker Apps

Participants’ affective responses and evaluations following their use of symptom checkers varied widely. While some participants expressed in the respective entries positive or satisfied feelings and evaluations, others reported negative or dissatisfied emotions and evaluations, and some were ambivalent.

Woke up with neck pain, according to [the SCA] it could be a tension. [The SCA] recommended heat and stretching, which helped. App usage easy, some questions were alarming.

Positive evaluations were associated with various facets of SCA usage. These include feelings of satisfaction and contentment, perceiving the information offered by the SCA as helpful and suitable, experiencing a heightened sense of security, achieving a better grasp of one’s symptoms, enriching the user’s knowledge, and finding the SCA’s responses to be logical, realistic, or plausible. Affective responses to SCA use encompass feeling calm, relieved, or more relaxed, feeling sensitized, and motivated to take action. Furthermore, users reported an increased sense of agency and empowerment.

I found it very interesting to read about this topic. I notice that the [SCA] is also helpful and useful to me, even for minor physical complaints.

Participants’ negative and dissatisfied emotions and evaluations were associated with several aspects of SCA use. Some participants reported having no trust in the symptom checker, finding the information provided unrealistic or unlikely, and feeling that the SCA worsened their situation if they were already experiencing health anxiety. Others found the information provided to be partially or entirely inaccurate or unhelpful, and some found the recommendations exaggerated or irritating. The negative emotions experienced by participants included disappointment, shock, disillusionment, or feeling unsure, anxious, scared, alarmed, or worried, and some expressed surprise or disappointment in the results provided by the SCA.

The [SCA] gives me three possible causes: tonsillitis, peritonsillar abscess, and retropharyngeal abscess. For all three, it recommends visiting the emergency room. I think it's exaggerated to go to the emergency room for a sore throat since I don't have a fever.

#### Behavioral Response Considering the Usage of Symptom Checker Apps

Participants reported various behaviors following their use of SCA. In some cases, participants searched the internet for more detailed information, while others preferred to wait and observe their symptoms before deciding whether to seek medical care. Self-treatment strategies were frequently mentioned, such as using over-the-counter medicine, increasing fluid intake, relaxing, or using home remedies.

I did not follow the advice of the [SCA] app to seek medical advice as I want to treat it myself with the ointment for a few days first.

In some cases, participants contacted the health care system and either considered making a doctor’s appointment, immediately made a doctor’s appointment, or decided to address their symptoms during their next scheduled appointment. However, some participants explicitly stated that they did not plan to discuss their SCA use with a doctor. A few participants made it clear that they chose not to follow the guidance provided by the SCA.

[The SCA] advises going to the emergency room, no possibility to see a doctor, was very worried throughout the day, wanted to go directly to the emergency room after the day, but symptoms have subsided a bit, so waited until the next day.

#### Unmet Requirements

Participants in this study identified a number of unmet requirements when using the SCA. Some participants expressed the need for a more complex symptom input system that allows for a more nuanced description of symptoms, as well as the ability to differentiate between different qualities and quantities of symptoms.

My symptoms were both diverse and unspecific - therefore [SCA] couldn't really make any concrete suggestions - as good as the help texts are, sometimes the questions asked are difficult to answer - I miss [the SCA] not knowing me better from the beginning, so I have fed her with pre-existing conditions, I am curious to see how well Ada learns from that. It's a shame that this cannot yet be taken into account in the suggestions.

Others reported that their symptoms were not recognized by the SCA and that there was no category for their specific complaint. Participants also expressed concern over the fact that symptoms and diagnoses (eg, bronchitis or cough) can be entered into the SCA. Participants expressed that the weighting of symptoms should be based on the leading symptom entered. Some participants found the questions difficult to answer and reported redundant questions. Participants requested a more differentiated classification of symptoms, as well as the ability to recognize different causes of multiple entered symptoms. Participants evaluated the level of personalization in the symptom checker as insufficient, as preexisting medical conditions, lifestyle, family history, and medication use were not considered.

Since I am young, exercise a lot, drink little, do not smoke, and generally live very healthily, I rarely had significant complaints. When something did occur, it was mostly self-inflicted (hangover feeling, sun, sports injuries). I would have wished that not only symptoms but also more lifestyle decisions had been addressed in the assessment. Most often, these explained my symptoms very accurately.

Some participants also requested the recognition of medication side effects and interactions, as well as consideration of lifestyle factors such as alcohol consumption and sleep deprivation. In addition, participants suggested that the SCA provide recommendations for home remedies and medication use. Participants also requested more educational material that illustrates the relationship between symptoms and specific advice. Finally, participants were concerned about the unclear source of the information provided by the SCA.

## Discussion

### Principal Results

This mixed methods study aimed to understand the patterns of SCA usage and to describe its use among medical laypersons. The new appearance of unfamiliar symptoms was found to be a strong predictor for SCA usage. In addition, specific ICPC symptom clusters such as “cardio-vascular,” “eye,” “skin,” “musculoskeletal,” and “general and unspecified symptoms” were also associated with a higher probability of SCA use. The qualitative data analysis identified 4 main themes reflecting the aspects that participants found noteworthy after using an SCA. The main themes comprised (1) reasons for using SCA, (2) diverse affective responses, (3) a broad spectrum of behavioral reactions, and (4) unmet needs including a lack of personalization.

### Comparison to Previous Work

To evaluate SCA use in a broader context it is worthwhile to reflect on why patients go to see a physician. Braunacker-Mayer and Avery [27] described that the decision to seek help from a physician depends on complex aspects of social, psychological, cultural, and biomedical factors. She described patients as “non-passive users of health care services”; who do not visit doctors without consideration. Further, she claims that patients evaluate their own health condition and then take steps to manage symptoms or treat illnesses. Thus, the decision-making process for seeking medical assistance may not align with clinicians’ perspectives, as it is influenced by a broader range of factors beyond biomedical considerations. With regard to the limited understanding of the SCA usage context, it seems beneficial to explore how the extended version of the Andersen model, a well-established framework in health care usage research, can provide insights into this aspect. The Andersen model [28] incorporates three dimensions to analyze the factors influencing health care usage. These dimensions include predisposing characteristics (such as age and gender), enabling resources (like access to health care facilities and income), and need factors (such as the prevalence of physical illnesses and perceived health status). A further study suggested expanding the model to include psychosocial factors, such as loneliness, and personality traits, like neuroticism [29]. In the following sections, we will examine and classify the identified predictors in the context of SCA usage and health care usage, with qualitative data being integrated subsequently. In the following paragraphs, we use the Andersen model on health care usage to contextualize our results.

#### Predictor “Initial Occurrence of Symptoms”

In this study, we found that the predictor “initial occurrence of symptoms” had a large effect on SCA use. In line with the Andersen model for health care usage [28] this predictor might represent a need factor. Furthermore, it can be considered as both situational and personal as the “initial occurrence of symptoms” relies on specific symptoms presented by an individual and includes the fact that this individual had experienced similar symptoms before or not. The perception of novel or familiar symptoms relies on prior experiences and the personal capacity to distinguish between different symptoms as well as the overall evaluation of symptoms. It is remarkable that other data have revealed anxiety as a predictor for SCA use in a cross-sectional setting [6]. Health anxiety might have an influence on the appraisal of “initially occurred symptoms” as relevant or irrelevant. Therefore, psychosocial factors or personality traits not only may have an influence on face-to-face health care usage but also on SCA use.

Through qualitative data analysis, we gained deeper insights into the underlying needs related to the predictor “initial occurrence of symptoms.” The category “purpose of use” in the qualitative results shed light on users’ motivations for using SCA. Users reported using these applications to gather information about medical conditions, symptoms, and treatment options. They also wanted to assess their own symptoms or those of close individuals to better understand potential underlying health issues. Another reason for using SCA was to determine whether a visit to a healthcare professional was necessary. These findings indicate that SCA contributes to the bricolage of health literacy. This perspective defines health literacy as a multidimensional concept, emerging from situational, dynamic, and social practices [30]. Health care bricolage involves the creative assembly and usage of a broad spectrum of resources, including a variety of knowledges, ideas, materials, and networks, to manage specific health concerns [31]. SCA seems to adapt and serve distinct functions, using diverse sources and types of health knowledge, along with the interplay between social connections and situational factors. They can be used by patients as a flexible resource tailored to their personal and situational needs, parallel to other resources.

#### Predictor “Self-Rated Health”

Compared to previous findings regarding positive associations between health care usage and self-rated health [32], self-rated health was identified as a predictor with a minor negative effect on SCA use. Regarding the association between need factors in the Andersen model [28] of health care usage, substantial evidence demonstrated a positive link between these factors [33]. In particular, self-rated health showed a strong association with health care usage. This finding is highly plausible since an increase in health care needs often indicates illness symptoms that prompt individuals to consult a physician, as supported by numerous cross-sectional studies. This relationship may not apply in the context of SCA use as they are a lower threshold offer and users’ needs are therefore less connected to illness symptoms and may be strongly connected to curiosity or insecurities raised by the initial appearance of symptoms and health anxiety and personality traits as mentioned above. In another cross-sectional study, self-rated health was examined in the context of inclination to use SCA, and a positive association was found [6]. Interestingly, no association was found between finding SCA useful and self-rated health [6]. Nevertheless, the recent findings concerning the relationship between SCA and self-rated health remain inconclusive, emphasizing the need for further research considering the inclination and the actual usage of SCA to delve into this aspect more comprehensively.

#### Predictor “Day of Measurement“

A further predictor with an uncertain role and ambiguous impact was the “day of measurement,” which indicated that the timing within the study significantly influenced SCA usage. However, the effect size of this predictor was too small to meaningfully influence the outcome. It should be noted that the large number of measurements may have contributed to the significance of the predictor.

#### Predictors Classified by International Classification in Primary Care

Other predictors examined were symptoms entered into the SCA categorized according to Symptoms of the categories “cardiovascular,” “general and unspecified,” “musculoskeletal,” “eye,” and “skin” were selected as relevant predictors, and all revealed a decent effect size and significant ORs. All named symptom clusters are reported as frequent reasons for emergency consultations in different studies [34-36].

The OR of the predictor “cardiovascular” symptoms was found to be the highest in our study, indicating that the usage of SCA is associated with potentially threatening symptoms. From a qualitative study in our project, it emerged that users would not use SCA in acute emergencies, such as a traumatic brain injury after a traffic accident. However, the findings suggest that users do use SCA for potentially severe and, therefore, anxiety-inducing symptoms. This could be explained by a form of internal triage by medical laypersons. In the case of an obviously nondeferrable emergency, SCA is not used. However, when uncertainties exist, and symptoms are anxiety-inducing but not immediately classifiable as time-sensitive and life-threatening, SCA is used. However, a study from 2022 [35] revealed that SCA does not exhibit a notable performance in such critical scenarios. This should be considered a high-risk situation for patients who may rely on SCA recommendations that are potentially wrong. “General and nonspecific” symptoms often point to a range of possible conditions, posing challenges in their attribution. Consequently, they could serve as meaningful predictors for SCA usage, as users seek additional information to determine the severity and urgency of such symptoms. This need for further clarification underscores the importance of SCA in assisting users to classify and understand their symptoms accurately.

The appearance of symptoms of the cluster “musculoskeletal,” “eye,” and “skin” frequently leads to a high level of distress as they are restricting in everyday life and therefore might lead to SCA usage. “Musculoskeletal” symptoms lead to movement restrictions and are therefore frequently perceived as constraining in everyday life. A study reports them as the main symptoms of attending an ED [36]. Symptoms concerning the eyes are often perceived as fear-inducing and irritating [37]. Moreover, symptoms concerning the skin are commonly visible and could therefore be stigmatizing [37]. They are also reported as symptoms with a low subjectively perceived treatment urgency that, however, lead patients to seek emergency consultations [34].

The identified ICPC symptom clusters are in accordance with the 10 most common general practitioner consultation reasons reported by the CONTENT project [38]. This may indicate 2 aspects: First, the need for low-threshold medical advice for these symptom clusters; Second SCAs are used for symptoms that may lead to contact with the medical system, and SCAs presumably have an impact on health care professionals and further resources.

### Strengths and Limitations

The study investigated the use of a specific SCA over a 42-day period and achieved a 0% dropout rate due to compensation, regular reminders, and user workshops. As a result, the data had less than 5% missing values. Since all participants used the same SCA to ensure comparability, our results may not be generalizable to all available SCAs. Due to the low frequencies of certain ICPC-2 chapters, some predictors were excluded, which may have resulted in some relevant but rarely occurring predictors being left out of the data. Another crucial aspect to consider with the ICPC-2 is its inability to reveal symptom severity or urgency. As a result, the recent findings do not take these factors into account. It should be noted that LASSO penalization in variable selection comes with certain general limitations. LASSO tends to favor sparse models over complex ones, resulting in models that are more interpretable [39]. However, in cases where there is a high degree of multicollinearity, this preference for sparsity can lead to instability in predictor selection [39]. While we anticipate some multicollinearity among predictors, particularly due to overlaps between ICPC-coded symptoms, overall, multicollinearity is not expected to be a significant issue. LASSO is generally effective at handling moderate multicollinearity [39]. In addition, variable selection in LASSO is influenced by the choice of the shrinking term and penalty [39], which represents an inherent limitation of the method. Nonetheless, compared with other variable selection techniques such as forward or backward selection, LASSO proves to be better suited for the presented data, particularly when considering moderate multicollinearity and the volume of data available.

Moreover, the sample consisted predominantly of females and younger adults, raising uncertainty about its representativeness of the entire user demographic or whether it reflects a sampling bias Nonetheless, this user sample aligns with other findings regarding the sociodemographic characteristics associated with SCA usage [6]. Consequently, additional studies encompassing broader user groups and diverse use cases are imperative to extrapolate the findings more universally.

### Conclusion

In this study, SCAs were more frequently used in situations characterized by greater uncertainty for example in situations when symptoms appeared for the first time. This suggests that the concept of intolerance to uncertainty may influence SCA use. Further research is essential to explore the relationships between intolerance of uncertainty, health anxiety, and SCA use as this aspect was not addressed in this study. In addition, the symptom clusters identified in our study had a large overlap with symptoms reported in emergency department visits considering recent study results, indicating that SCA usage could potentially affect health care usage. Further research is needed to ascertain whether such effects exist and how they impact health care usage. Moreover, SCAs are not only used intentionally and purposefully; they are also used playfully, for reasons beyond personal use and as this study showed to validate the user's or doctor’s hypotheses. Furthermore, they are used to verify the accuracy of the software itself, especially when a diagnosis is already known. This reveals that SCAs contribute to a broader bricolage of health literacy, serving as one of many tools integrated into a wider context of sources of information regarding one’s health. Given the current low usage rate of SCAs in Germany, this effect may though be minor at present in the German health care system. Nevertheless, various stakeholders in the German health care system have shown interest in SCAs. Currently, it remains uncertain how this interest will affect SCA usage rates in the future.

## Appendix

Multimedia Appendix 1 Execution for the generalized linear mixed models.

Multimedia Appendix 2 Overview of the missings.

Multimedia Appendix 3 Overview of the symptom checker app use or nonuse cases considering potential contributing variables.

Multimedia Appendix 4 International Classification in Primary Care 2–coded symptoms stratified for symptom checker app use or nonuse.

Multimedia Appendix 5 Binned residual plot for Model 1-3.

## Abbreviations

BIC: Bayesian information criterion
GLMM: Generalized linear mixed model
ICPC: International Classification in Primary Care 2
ISO: International Organization for Standardization
LASSO: least absolute shrinkage and selection operator
SCA: symptom checker apps
STROBE: Strengthening the Reporting of Observational Studies in Epidemiology

## Data Availability

The datasets generated during and/or analyzed during this study are available from the corresponding author on reasonable request.
