# Supplementary material for: Unveiling Usage Patterns and Explaining Usage of Symptom Checker Apps: Explorative Longitudinal Mixed Methods Study
Source: J Med Internet Res. 2024 Dec 9;26:e55161. doi: 10.2196/55161 (PMC11667141; doi:10.2196/55161)
Supplement: Multimedia Appendix 1 [file jmir_v26i1e55161_app1.docx]

## Execution for the General Linear Mixed Models

To consider the hierarchical data with more than one level General Linear Mixed Models (GLMM) with and without regularization were fit to the data and compared.

In the present work, a LASSO (**L**east **A**bsolute **S**hrinkage and **S**election **O**perator, ^1^) regularization was applied to a GLMM to derive meaningful fixed and random effects. The LASSO penalty has the advantage that it shrinks parameters to exactly zero when the tuning parameter λ is sufficiently large ^2^. The R package glmmPen was applied to the data and the best fitting model chosen considering the Bayesian Information Criterion. The parameters of the model were estimated by using an Monte Carlo Expectation Conditional Maximization (MCECM) algorithm, which leverages Stan and RcppArmadillo to increase computational efficiency ^2^. The glmmPen function also offers automated tuning parameter selection and automated initialization of the random effect variance ^2^.

Model assessment was realized by using the BIC (Bayesian Information Criterion) Scores of the Models. The BIC is a widely used score that prefers more parsimonious models over complex models. Lower values of the BIC show a better model performance ^3^.

1. Tibshirani R. Regression shrinkage and selection via the lasso. *Journal of the Royal Statistical Society: Series B (Methodological)* 1996; 58: 267-288.

2. Heiling H. RN, Li Q., Ibrahim J. glmmPen: High Dimensional Penalized Generalized Linear Mixed Models (pGLMM). 2021.

3. Schwarz G. Estimating the dimension of a model. *The annals of statistics* 1978: 461-464.
