# Supplementary material for: Unveiling Usage Patterns and Explaining Usage of Symptom Checker Apps: Explorative Longitudinal Mixed Methods Study
Source: J Med Internet Res. 2024 Dec 9;26:e55161. doi: 10.2196/55161 (PMC11667141; doi:10.2196/55161)
Supplement: Multimedia Appendix 2 [file jmir_v26i1e55161_app2.docx]

## Overview of the Missings

Variable description Scale level Missings (n)

Age continuous 0

Gender binary 0

Day of study integer 0

First time appearance of Symptoms binary 0

Subjective rated health interval 9

Subjective rated mood interval 12

Subjective rated stress interval 7

Symptom cluster categorized by ICPC 14 dummy coded variables 0
