# Supplementary material for: Unveiling Usage Patterns and Explaining Usage of Symptom Checker Apps: Explorative Longitudinal Mixed Methods Study
Source: J Med Internet Res. 2024 Dec 9;26:e55161. doi: 10.2196/55161 (PMC11667141; doi:10.2196/55161)
Supplement: Multimedia Appendix 3 [file jmir_v26i1e55161_app3.docx]

Overview of the SCA (non-)use cases considering potential contributing variables

Variables no Ada use Ada use Total p CI (N=1743) (N=262) (N=2005)

**Medication intake**

| no | 1534 (89%) | 179 (69%) | 1713 (86%) | <0.001 ^chi2^ | [0.14, 0.25]^PWa^ |
| --- | --- | --- | --- | --- | --- |
| yes | 189 (11%) | 81 (31%) | 270 (14%) |  |  |
| (Missing) | 20 | 2 | 22 |  |  |

**Subjective rated**

<0.001

| **health**  very bad | 10 (1%) | 10 (4%) | 20 (1%) |
| --- | --- | --- | --- |
| bad | 85 (5%) | 44 (17%) | 129 (6%) |
| partly | 393 (23%) | 125 (48%) | 518 (26%) |
| good | 881 (51%) | 60 (23%) | 941 (47%) |
| very good | 371 (21%) | 20 (8%) | 391 (20%) |
| (Missing) | 3 | 3 | 6 |

chi2

**Subjective rated**

| **mood**  very bad | 27 (2%) | 7 (3%) | 34 (2%) |
| --- | --- | --- | --- |
| bad | 125 (7%) | 40 (15%) | 165 (8%) |
| partly | 395 (23%) | 95 (36%) | 490 (25%) |
| good | 841 (48%) | 93 (36%) | 934 (47%) |
| very good | 348 (20%) | 26 (10%) | 374 (19%) |
| (Missing) | 7 | 1 | 8 |

**Subjective rated mood**

| 601 (35%) | 59 (23%) | 660 (33%) |
| --- | --- | --- |
| 857 (49%) | 142 (54%) | 999 (50%) |
| 282 (16%) | 60 (23%) | 342 (17%) |
| 3 | 1 | 4 |

not stressed at

all

a bit stressed very stressed (Missing)

<0.001^chi2^

<0.001^chi2^

| **Symptoms**  **appeared for the** |  | | | | | | |
| --- | --- | --- | --- | --- | --- | --- | --- |
| **first time**  no | 1688 (97%) | 187 (71%) | 1875 (94%) | <0.001 | chi2 | [0.39, 0.56] | PWa |
| yes | 55 (3%) | 75 (29%) | 130 (6%) |  |  |  |  |

chi2 Pearson’s chi-squared test

PWa CI for difference in proportions derived from a normal “Wald” approximation
