# Supplementary material for: Unveiling Usage Patterns and Explaining Usage of Symptom Checker Apps: Explorative Longitudinal Mixed Methods Study
Source: J Med Internet Res. 2024 Dec 9;26:e55161. doi: 10.2196/55161 (PMC11667141; doi:10.2196/55161)
Supplement: Multimedia Appendix 4 [file jmir_v26i1e55161_app4.docx]

## ICPC coded symptoms stratified for SCA (non)-use.

Variables no Ada use Ada use Total p CI (N=1743) (N=262) (N=2005)

**General and**

| **unspecified**  present | 306 (18%) | 176 (67%) | 482 (24%) | <0.001chi2 | [-0.35, -0.26]PWa |
| --- | --- | --- | --- | --- | --- |
| not present | 1437 (82%) | 86 (33%) | 1523 (76%) |  |  |

**Blood**

| present | 1 (0%) | 2 (1%) | 3 (0%) | 0.006chi2 | [-1, -0.0032]PWa |
| --- | --- | --- | --- | --- | --- |
| not present | 1742 (100%) | 260 (99%) | 2002 (100%) |  |  |

**Digestive**

| present | 91 (5%) | 54 (21%) | 145 (7%) | <0.001chi2 | [-0.34, -0.18]PWa |
| --- | --- | --- | --- | --- | --- |
| not present | 1652 (95%) | 208 (79%) | 1860 (93%) |  |  |

**Eye**

| present | 10 (1%) | 14 (5%) | 24 (1%) | <0.001chi2 | [-0.66, -0.26]PWa |
| --- | --- | --- | --- | --- | --- |
| not present | 1733 (99%) | 248 (95%) | 1981 (99%) |  |  |

**Ear**

| present | 6 (0%) | 4 (2%) | 10 (0%) | 0.011chi2 | [-0.57, 0.033]PWa |
| --- | --- | --- | --- | --- | --- |
| not present | 1737 (100%) | 258 (98%) | 1995 (100%) |  |  |

**Cardiovascular**

| present | 15 (1%) | 14 (5%) | 29 (1%) | <0.001chi2 | [-0.54, -0.17]PWa |
| --- | --- | --- | --- | --- | --- |
| not present | 1728 (99%) | 248 (95%) | 1976 (99%) |  |  |

**Musculosketal**

| present | 425 (24%) | 211 (81%) | 636 (32%) | <0.001chi2 | [-0.33, -0.26]PWa |
| --- | --- | --- | --- | --- | --- |
| not present | 1318 (76%) | 51 (19%) | 1369 (68%) |  |  |

**Neurological**

| present | 193 (11%) | 78 (30%) | 271 (14%) | <0.001chi2 | [-0.24, -0.13]PWa |
| --- | --- | --- | --- | --- | --- |
| not present | 1550 (89%) | 184 (70%) | 1734 (86%) |  |  |

**Psychological**

| present | 51 (3%) | 25 (10%) | 76 (4%) | <0.001chi2 | [-0.31, -0.099]PWa |
| --- | --- | --- | --- | --- | --- |
| not present | 1692 (97%) | 237 (90%) | 1929 (96%) |  |  |

**Respiration**

| present | 40 (2%) | 25 (10%) | 65 (3%) | <0.001chi2 | [-0.38, -0.14]PWa |
| --- | --- | --- | --- | --- | --- |
| not present | 1703 (98%) | 237 (90%) | 1940 (97%) |  |  |

**Skin**

| present | 16 (1%) | 26 (10%) | 42 (2%) | <0.001chi2 | [-0.65, -0.35]PWa |
| --- | --- | --- | --- | --- | --- |
| not present | 1727 (99%) | 236 (90%) | 1963 (98%) |  |  |

*(continued)*

Variables no Ada use Ada use Total p CI (N=1743) (N=262) (N=2005)

**Endocrine**

| present | 15 (1%) | 15 (6%) | 30 (1%) | <0.001chi2 | [-0.55, -0.2]PWa |
| --- | --- | --- | --- | --- | --- |
| not present | 1728 (99%) | 247 (94%) | 1975 (99%) |  |  |

**Urology**

| present | 41 (2%) | 19 (7%) | 60 (3%) | <0.001chi2 | [-0.31, -0.073]PWa |
| --- | --- | --- | --- | --- | --- |
| not present | 1702 (98%) | 243 (93%) | 1945 (97%) |  |  |

**Female genital**

| **system**  present | 40 (2%) | 10 (4%) | 50 (2%) | 0.141chi2 | [-0.18, 0.041]PWa |
| --- | --- | --- | --- | --- | --- |
| not present | 1703 (98%) | 252 (96%) | 1955 (98%) |  |  |

chi2 Pearson’s chi-squared test

PWa CI for difference in proportions derived from a normal (”Wald”) approximation
