# Supplementary material for: Unveiling Usage Patterns and Explaining Usage of Symptom Checker Apps: Explorative Longitudinal Mixed Methods Study
Source: J Med Internet Res. 2024 Dec 9;26:e55161. doi: 10.2196/55161 (PMC11667141; doi:10.2196/55161)
Supplement: Multimedia Appendix 5 [file jmir_v26i1e55161_app5.docx]

## Binned residual plot for Model 1-3

0.0 0.2 0.4 0.6 0.8


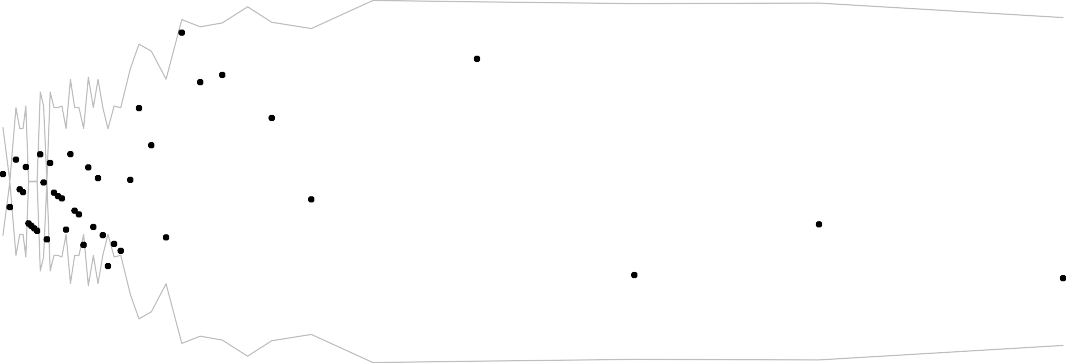


Average residual

−0.15 −0.10 −0.05 0.00

0.05

Expected Values

Binned residual plot Model 1

0.15

0.0 0.2 0.4 0.6 0.8


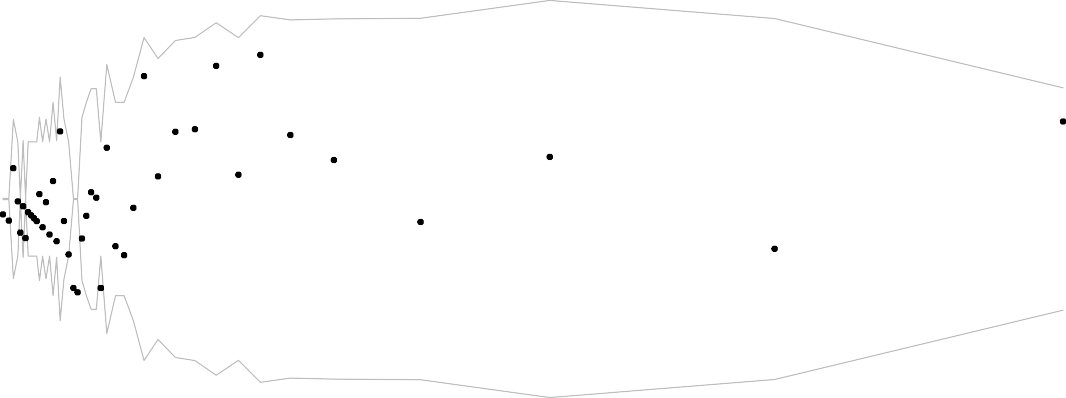


Average residual

−0.15 −0.10 −0.05

0.00

Expected Values

Binned residual plot Model 2


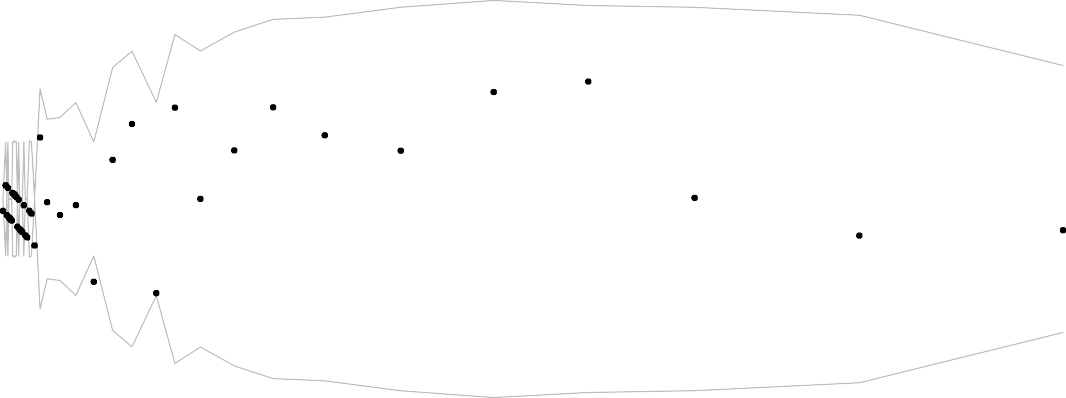


0.05

0.10

0.15

0.0 0.2 0.4 0.6 0.8

Average residual

−0.15 −0.10 −0.05

0.00

Expected Values

Binned residual plot Model 3
